# Pedestrian motion modelled by Fokker–Planck Nash games

**DOI:** 10.1098/rsos.170648

**Published:** 2017-09-13

**Authors:** S. Roy, A. Borzì, A. Habbal

**Affiliations:** 1 Institut für Mathematik, Universität Würzburg, Emil-Fischer-Strasse 30, 97074 Würzburg, Germany; 2 Université Côte d'Azur, Inria, CNRS, LJAD, UMR 7351, Parc Valrose, 06108 Nice, France

**Keywords:** pedestrian motion, avoidance, Fokker–Planck equation, differential games, Nash equilibrium, optimal control

## Abstract

A new approach to modelling pedestrians' avoidance dynamics based on a Fokker–Planck (FP) Nash game framework is presented. In this framework, two interacting pedestrians are considered, whose motion variability is modelled through the corresponding probability density functions (PDFs) governed by FP equations. Based on these equations, a Nash differential game is formulated where the game strategies represent controls aiming at avoidance by minimizing appropriate collision cost functionals. The existence of Nash equilibria solutions is proved and characterized as a solution to an optimal control problem that is solved numerically. Results of numerical experiments are presented that successfully compare the computed Nash equilibria to the output of real experiments (conducted with humans) for four test cases.

## 1. Introduction

Multiple pedestrian motion is a complex social and biological process [1,2], involving psychological and non-deterministic behavioural decisions. It includes features like pattern formation, e.g. groups and lanes, and non-rational dynamics as in a panic situation. A good knowledge of a pedestrian and, more generally, crowd flow scenario is of utmost importance for urban management and safe evacuation [3,4], hence the importance of realistic modelling and simulation, notably with the help of mathematical and computational tools.

There is a huge literature dedicated to the modelling of crowd motion, starting from original studies in the late 1950s [5], with the early paper [6] where fluid mechanics models are proposed, and the paper [7] where pedestrians are modelled as interacting particles with mechanical attractive–repulsive forces. Presently, pedestrian modelling encompasses mathematical approaches ranging from discrete and cellular automata to continuum fluid dynamics, conservation laws and hybrid approaches [1,8–14].

**Figure 1.**
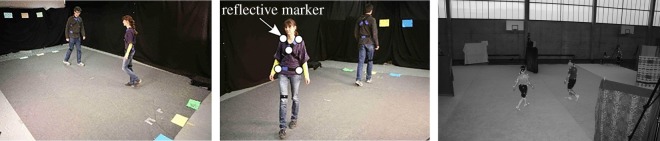
Two avoidance experimentsinvolving humans: the first one (images left and centre) is due to Turnwald *et al.* [18]. In this experiment, the pedestrians have early and continuous visual information on the other's moves, appealing for dynamic decision-making. The second one (right B&W image), referred to as Gait and Posture in our experiments section, is due to Olivier *et al.* [17]. Blankets are used to hide each pedestrian's early moves, allowing for exchange of information only at a late time-to-see, so that the interaction between the two players/pedestrians could be modelled as a static game, more precisely a static Nash game.

In this framework, *avoidance* is one of the most important and challenging features in pedestrian motion [15], involving non-cooperative and possibly non-local interactions. Understanding avoidance mechanisms has attracted much attention from experimentalists [16–18]. However, despite the obvious relevance of a game-theoretic framework, to the best of our knowledge only a very few publications are dedicated to the investigation of this fundamental process. Let us mention the study [18] where human experiments are led to assess the interaction-based decision-making involved in the avoidance behaviour (figure 1), and the study [11] where the modelling of deterministic pedestrian flow within an ODE optimal control and differential games framework is studied. There is also an important literature focusing on mean field games approaches to model pedestrian motion, see, for instance [19] and the references therein. These approaches do, however, basically assume a very large number of agents or of interacting particles [20].

In this paper, we introduce a new approach that starts from the framework developed in [21], where (open- and closed-loop) optimal control of crowd motion in the framework of stochastic processes and the related Fokker–Planck (FP) equations is discussed, and considers *two* individuals, who behave with rationality (in the game vocabulary sense) and have enough motion variability to be suitably described by the probability density functions (PDFs) of their motions. Specifically, the dynamics of the two pedestrians are assumed to be stochastic processes modelled by stochastic differential equations (SDEs), each with a drift composed of a desired velocity (related to a desired path) and a control function. Further, a Wiener process is included to model dispersal due to e.g. variability among individuals or in pavement roughness. In this modelling context, we refer the reader to the study Hoogendorn & Bovy [12] for an earlier work considering a single pedestrian modelled by an SDE.

Corresponding to these SDEs, we consider the related FP equations that model the evolution of the PDFs of the state (position) of the pedestrian. These FP equations are parabolic convection–diffusion equations where the convective coefficient corresponds to the drift of the SDE and thus inherits the control mechanism, which represents the players' strategy for avoidance. On the other hand, a main contribution of our work is the formulation of functional objectives that appropriately model the chance of collision that needs to be minimized by the action of the controls and subject to the differential constraints given by the FP equations of the two pedestrians. Our next essential step is the formulation of the avoidance problem as a static Nash game with complete information such that the avoidance dynamics arises as a Nash equilibrium (NE) of the game. We remark that, in our framework, the concept of NE for modelling the decision-making control for collision avoidance plays a central role, and results in the formulation of a partial-differential open-loop Nash game governed by FP equations where the control is included in the drift of the stochastic pedestrian motion, and cost functionals for each pedestrian are defined that include a cost of the control and a collision-penalizing term.

We prove the existence of optimal Nash equilibria solutions and investigate the regularity of these solutions. For the numerical simulation of the proposed partial-differential games, an approximation and optimization framework for the fast computation of Nash solutions is discussed. The resulting FP-Nash computational framework for pedestrian motion is benchmarked with results of real experiments from cognitive psychology studies. An important feature of the present approach is that the pedestrian dynamics arise as a result—an NE—of interaction with prescribed rules, while in classical approaches the dynamics are prescribed. In other words, the present game approach explains *why* the observed dynamics arise, while usual phenomenological approaches [1,8–10,14] prescribe *how* the dynamics should be. However, further developments may reasonably witness a merging of our non-mechanistic FP-Nash modelling of avoidance with additional features as social rules and forces.

In the next section, we formulate our FP-Nash game model for avoidance. On the one hand, we motivate the modelling of the positions of pedestrians (the players) with stochastic differential models and with the corresponding FP equations. On the other hand, in the framework of differential games, we introduce into each SDE, and correspondingly FP, a control function that represents the strategy of the player with the purpose of optimizing its own objective. In §3, these objectives are formulated in terms of the PDFs of the state of the pedestrian and consist of a terminal term modelling a desired final position, a control cost and a collision functional. Further, the avoidance problem is defined as an NE problem and its solution is characterized as the minimum of a composite reduced cost functional. In §4, the solution of this composite minimization problem is framed as a control-constrained optimal control problem governed by the two FP equations corresponding to the two pedestrians. For this problem, we prove the existence of an optimal solution and discuss its characterization as the solution of an FP optimality system involving the two FP equations and the corresponding FP adjoint equations and optimality conditions. In §5, we carefully illustrate the setting and outcome of real experiments from cognitive psychology studies [16–18] that serve as a benchmark for the results of our FP-Nash avoidance framework. Specifically, we consider four test cases where avoidance is observed with different dynamical and geometrical settings. For these test cases, we aim at reproducing a similar setting in our FP-Nash computational scheme and compare our results (trajectories) with those that have been recorded in the real experiments. The similarity between our results and the output of the real experiments with human behaviour is impressive and supports the validity of our approach. A section of conclusion completes this work.

## 2. Formulation of the Nash game

Mathematical games may be static or dynamic. Roughly speaking, in a dynamic game, players sequentially observe the others' actions and then choose their optimal responses. In a static game, players choose their best responses to the others without exchange (or communication) of information. Games may also be with complete information, meaning that all players know each others' strategy spaces and cost functionals (including their own ones). The failure of this assumption is termed as a game with incomplete information, see [22] for details.

We model our differential game *as static with complete information*. We consider P∈N players, *P*≥2, that evolve according to *P* SDEs driven by *P* time-dependent control functions. Specifically, the states of our players are denoted with *X*^(*p*)^, 1≤*p*≤*P*, and belong to a space domain $\Omega \subset R^{D}$ ($D\in N^{*}$), which is assumed to be convex with Lipschitz boundary. We assume that the states of the players are subject to reflecting barriers, which may represent the walls of a room where the motion takes place. The time evolution of *X*^(*p*)^ is governed by the following SDE:

2.1 $$\begin{array}{rl} dX^{\left(p\right)}(t) & \;=b^{\left(p\right)}(X^{\left(p\right)}(t),t,u^{\left(p\right)}(t))\;dt+\sigma \;dB^{\left(p\right)}(t)\\ \text{and}\;X^{\left(p\right)}(0) & \;=X_{0}^{\left(p\right)}, \end{array}\}$$

where the drifts *b*^(*p*)^(*X*^(*p*)^(*t*),*t*,*u*^(*p*)^(*t*)) have the following structure:

2.2 $$b^{\left(p\right)}(X^{\left(p\right)}(t),t,u^{\left(p\right)}(t))=v^{\left(p\right)}(X^{\left(p\right)}(t),t)+u^{\left(p\right)}(t).$$

The velocity fields *v*^(*p*)^ represent the deterministic dynamics of the single players in the absence of interaction with other players. On the other hand, the Brownian process with a constant dispersion coefficient *σB*^(*p*)^(*t*) is included to model dispersal due to external physical forces or other perturbations to a deterministic motion (e.g. rough pavement, drunkenness). Indeed, the dispersion *σ* may depend on the state *X*^(*p*)^ and on time, but this is not essential for our modelling purpose.

The dynamics modelled by the drift *b* may represent the optimal (or preferred) path to satisfy the player's objective, e.g. to reach in the shortest time an arrival point *A*^(*p*)^ from a departure one *D*^(*p*)^, where *D*^(*p*)^ and *A*^(*p*)^ belong to *Ω*. In the drift, the time-dependent control functions *u*^(*p*)^ represent the strategy that the players *X*^(*p*)^ choose in order to satisfy the original objectives as much as possible, taking into account the presence of the other players. In mathematical terms, the players' objectives can be formulated as follows:

2.3 $$J_{p}(u^{\left(p\right)},u^{\left(-p\right)})=\alpha E[V_{p}(X^{p}(T)-X_{T}^{\left(p\right)})]+\frac{\nu }{2}∥u^{\left(p\right)}{∥}_{H^{1}(0,T;R^{D})}^{2}+W_{p}(X^{\left(p\right)},X^{\left(-p\right)}),$$

where the potential *V*_*p*_ denotes a convex function of the state with minimum at $X_{T}^{\left(p\right)}$, which denotes the desired final state, e.g. an exit point, of the player *p* and the superscript (−*p*) is used to emphasize the variables of player *p* and subsumes all the other players' variables. In (2.3), we also have an *H*^1^ cost of the control, with weight *ν*>0, which guarantees a bounded control effort and a continuous slow-varying control. Note that similar to [12], the first term in (2.3) can be interpreted as a benefit function. The term *W*_*p*_ is a key element of our work and it aims at modelling the interaction between players.

As shown in previous works [23,21], a convenient framework that accommodates the minimization of (2.3) subject to the SDE constraints given by (2.1) originates from the observation that the entire statistics of the stochastic process, modelled by *X*^(*p*)^(*t*), is characterized by the PDF of this process. In particular, the PDF describes in a natural way the probability distribution of the initial state configuration $X_{0}^{\left(p\right)}$ (or our uncertain knowledge of it). We denote this initial PDF by $f_{0}^{\left(p\right)}(x)$. Then, the evolution of the PDF *f*^(*p*)^=*f*^(*p*)^(*x*,*t*) of the state *X*^(*p*)^ at time *t* is governed by the following FP equations, 1≤*p*≤*P*,

2.4 $$\partial_{t}f^{\left(p\right)}(x,t)-\frac{\sigma^{2}}{2}\sum_{i=1}^{D}\partial_{x_{i}x_{i}}^{2}\;f^{\left(p\right)}(x,t)+\sum_{i=1}^{D}\partial_{x_{i}}(b_{i}^{\left(p\right)}(x,t,u_{i}^{\left(p\right)}(t))\;f^{\left(p\right)}(x,t))=0$$

and

2.5 $$f^{\left(p\right)}(x,0)=f_{0}^{\left(p\right)}(x).$$

This problem is considered in the space–time domain *Q*=*Ω*×(0,*T*). In this formulation, the initial vector PDF distribution satisfies the following conditions:

2.6 $$f_{0}^{\left(p\right)}\geq 0,\;\int_{\Omega }f_{0}^{\left(p\right)}(x)\;dx=1.$$

Corresponding to reflecting barriers for the stochastic model (2.1), we have flux zero boundary conditions for the FP equations. In order to formulate these boundary conditions, note that the FP problem (2.4) and (2.5) can be written in flux form as follows:

2.7 $$\partial_{t}f^{\left(p\right)}(x,t)=\nabla \cdot F^{\left(p\right)},\;f^{\left(p\right)}(x,0)=f_{0}^{\left(p\right)}(x),$$

where ‘∇⋅’ denotes the divergence operator and the flux *F*^(*p*)^ is given component-wise by

$$F_{j}^{\left(p\right)}(x,t;f)=\frac{\sigma^{2}}{2}\partial_{x_{j}}f^{\left(p\right)}-b_{j}^{\left(p\right)}(x,t,u_{j}^{\left(p\right)}(t))f^{\left(p\right)}.$$

Therefore, flux zero boundary conditions are formulated as follows:

2.8 $$F^{\left(p\right)}\cdot n=0,\;\text{on }\partial \Omega \times (0,T),$$

where *n* is the unit outward normal on ∂*Ω*.

As discussed in [21], for a given *u*^(*p*)^∈*H*^1^(0,*T*) and $f_{0}^{\left(p\right)}\in H^{1}(\Omega )$ there exists a unique solution *f*^(*p*)^∈*C*([0,*T*];*H*^1^(*Ω*)) to (2.4)–(2.8), and the control-to-state maps *u*^(*p*)^↦*f*^(*p*)^, 1≤*p*≤*P*, are differentiable.

In the FP framework, the players' objectives (2.3) can be reformulated as follows:

2.9 $$J_{p}(u^{\left(p\right)},u^{\left(-p\right)})=\alpha \int_{\Omega }V_{p}(x-x_{T}^{\left(p\right)})f^{\left(p\right)}(x,T)\;dx+\frac{\nu }{2}∥u^{\left(p\right)}{∥}_{H^{1}(0,T;R^{D})}^{2}+W_{p}(\;f^{\left(p\right)},f^{\left(-p\right)}).$$

Note that, because by the control-to-state map the PDF is a function of the control strategy, we may choose that the PDF functions do not appear as an argument of the objectives *J*_*p*_, which is then known as the reduced cost functional.

Our novel modelling step is the construction of the interaction functional *W*_*p*_( *f*^(*p*)^,*f*^(−*p*)^). For this purpose and for clarity of our discussion, the remaining part of the paper is devoted to the case *P*=2 of two pedestrians/players. However, our results can be extended to the more general case of *P*>2.

Now, we start defining *W*_*p*_( *f*^(1)^,*f*^(2)^) as a statistical expectation of the following general form:

2.10 $$W_{p}(\;f^{\left(1\right)},f^{\left(2\right)})=\int_{0}^{T}E\{Q_{p}(t,X^{\left(1\right)}(t),X^{\left(2\right)}(t),u^{\left(1\right)}(X^{\left(1\right)}(t),t),u^{\left(2\right)}(X^{\left(2\right)}(t),t))\}\;dt$$

or

2.11 $$\max_{t\leq T}E\{Q_{p}(t,X^{\left(1\right)}(t),X^{\left(2\right)}(t),u^{\left(1\right)}(X^{\left(1\right)}(t),t),u^{\left(2\right)}(X^{\left(2\right)}(t),t))\}.$$

However, classical variants of this formulation are possible (like considering the maximum of $E(Q_{p})$ over some prescribed schedules *t*∈[*t*_*i*_,*t*_*i*+1_]).

Regarding crowd behaviour, apart from avoiding obstacles and seeking for optimal routes, we make the obvious assumption that two pedestrians would prefer to avoid being in the same space location at the same time. Therefore, let us denote by *r*>0 the overcrowding limit in the sense that the two pedestrians above would avoid a situation where |*X*^(2)^(*t*)−*X*^(1)^(*t*)|<*r*, where |⋅| denotes e.g. the euclidean norm. Thus, we may consider that the two players are aimed at minimizing the probability of such an event and we set $\mathrm{Prob}\{|X^{\left(2\right)}(t)-X^{\left(1\right)}(t)|<r\}=E(Q_{p})$, where *Q*_*p*_ is the following characteristic function:

$$Q_{p}(X^{\left(1\right)}(t),X^{\left(2\right)}(t))={\text{1}}_{\left\{|X^{\left(2\right)}(t)-X^{\left(1\right)}(t)|<r\right\}}.$$

Owing to the independence of the two stochastic processes, *X*^(1)^(*t*) and *X*^(2)^(*t*), and assuming that *r* is small enough, we have

2.12 $$\begin{array}{r} E(Q_{p})=\int_{\Omega }\int_{\Omega }{\text{1}}_{|y-x|<r}f^{\left(1\right)}(x,t)f^{\left(2\right)}(y,t)\;dx\;dy \end{array}$$

2.13 $$\begin{array}{r} =\int_{\Omega }f^{\left(1\right)}(x,t)\int_{B(x,r)}f^{\left(2\right)}(y,t)\;dy\;dx\approx r^{D}\int_{\Omega }f^{\left(1\right)}(x,t)f^{\left(2\right)}(x,t)\;dx, \end{array}$$

where *B*(*x*,*r*) denotes the open ball |*y*−*x*|<*r* in $R^{D}$. The Fubini and averaging operations are licit because *f*^(1)^(⋅,*t*) and *f*^(2)^(⋅,*t*) are smooth enough as being solutions to the FP equations (given some assumptions; see Theorem 1 in [23]).

Motivated by (2.13), we choose the interaction cost *W*_*p*_( *f*^(1)^,*f*^(2)^) as follows:

2.14 $$W_{p}(\;f^{\left(1\right)},f^{\left(2\right)})=\rho \int_{\Omega }f^{\left(1\right)}(t,x)f^{\left(2\right)}(t,x)\;dx,$$

where the parameter *ρ* is defined as *ρ*=*Cr*^*D*^, and *C*≥0 denotes the relative strength of the interaction, tuned to balance the weights of the other terms in the cost functionals *J*_*p*_ in (2.9). As we consider a symmetric interaction for both players, we omit the index *p* in *W*_*p*_ in the discussion that follows.

## 3. Nash equilibrium

In this section, we formulate our differential game whose solution is sought as an NE. We discuss the characterization of this equilibrium solution and prove its existence.

We state our two pedestrian differential game as follows: The aim of pedestrian 1 is to choose strategy *u*^(1)^ to minimize

G1 $$J_{1}(u^{\left(1\right)},u^{\left(2\right)})=\alpha \int_{\Omega }V_{1}(x-x_{T}^{\left(1\right)})f^{\left(1\right)}(x,T)\;dx+\frac{\nu }{2}∥u^{\left(1\right)}{∥}_{H^{1}(0,T;R^{D})}^{2}+\rho \int_{0}^{T}\int_{\Omega }f^{\left(1\right)}(x,t)f^{\left(2\right)}(t,x)\;dx\;dt,$$

while pedestrian 2 aims at minimizing the following functional with strategy *u*^(2)^ as follows:

G2 $$J_{2}(u^{\left(1\right)},u^{\left(2\right)})=\alpha \int_{\Omega }V_{2}(x-x_{T}^{\left(2\right)})f^{\left(2\right)}(x,T)\;dx+\frac{\nu }{2}∥u^{\left(2\right)}{∥}_{H^{1}(0,T;R^{D})}^{2}+\rho \int_{0}^{T}\int_{\Omega }f^{\left(1\right)}(x,t)f^{\left(2\right)}(t,x)\;dx\;dt,$$

where *f*^(*p*)^, *p*=1,2, satisfy the FP problem (2.4) and (2.5). However, as both objectives depend on both strategies, we assume that the players decide to pursue an NE solution to this game.

Let *U*^(*p*)^ be the space of admissible strategies, *u*^(*p*)^∈*U*^(*p*)^. Then, an NE is defined as a pair of strategies $({\bar{u}}^{\left(1\right)},{\bar{u}}^{\left(2\right)})\in U^{\left(1\right)}\times U^{\left(2\right)}$ such that the following holds:

3.1 $$\begin{array}{r} \left({\bar{u}}^{\left(1\right)},{\bar{u}}^{\left(2\right)})=\arg\min_{u^{\left(1\right)}\in U^{\left(1\right)}}J_{1}(u^{\left(1\right)},{\bar{u}}^{\left(2\right)}\right) \end{array}$$

3.2 $$\begin{array}{r} =\arg\min_{u^{\left(2\right)}\in U^{\left(2\right)}}J_{2}({\bar{u}}^{\left(1\right)},u^{\left(2\right)}) \end{array}$$

Note that our objectives are not convex and therefore we cannot apply Nash's theorem [24] to state the existence of an NE. On the other hand, by exploiting the structure of our differential game, namely the weak coupling, we prove the existence of a Nash equilibrium solution in the following way: first, we show that solutions to a specific control problem are Nash equilibria of our game. Then, in the next section, we prove the existence of solutions to this optimal control problem.

Let us define

$$G_{p}(u^{\left(p\right)})=\alpha \int_{\Omega }V_{p}(x-x_{T}^{\left(p\right)})f^{\left(p\right)}(x,T)\;dx+\frac{\nu }{2}∥u^{\left(p\right)}{∥}_{H^{1}(0,T;R^{D})}^{2}.$$

With this notation, we have the following reduced objectives:

3.3 $$J_{p}(u^{\left(1\right)},u^{\left(2\right)})=G_{p}(u^{\left(p\right)})+W(u^{\left(1\right)},u^{\left(2\right)}).$$

In fact, with our setting, we have a separable game of the following form:

3.4 $$\begin{array}{rl} & \mathrm{Player}\;(1):\;J_{1}(u^{\left(1\right)},u^{\left(2\right)})=G_{1}(u^{\left(1\right)})+W(u^{\left(1\right)},u^{\left(2\right)})\\ \text{and}\; & \mathrm{Player}\;(2):\;J_{2}(u^{\left(1\right)},u^{\left(2\right)})=G_{2}(u^{\left(2\right)})+W(u^{\left(1\right)},u^{\left(2\right)}). \end{array}\}$$

Note that if the pedestrians are not too sensitive to overcrowding, then the present game belongs to the family of weakly coupled games and has a very useful separable structure. In the limit case where *ρ*=0, there is no game taking place, and we get only two independent single decision-making control problems.

Now, we define the following composite cost functional:

3.5 $$\hat{J}(u^{\left(1\right)},u^{\left(2\right)})=G_{1}(u^{\left(1\right)})+G_{2}(u^{\left(2\right)})+W(u^{\left(1\right)},u^{\left(2\right)})$$

and consider the optimal control problem

$$\min\hat{J}(u^{\left(1\right)},u^{\left(2\right)}),\;(u^{\left(1\right)},u^{\left(2\right)})\in U^{\left(1\right)}\times U^{\left(2\right)}.$$

In the following theorem, we prove that a solution to this optimal control problem is a Nash equilibrium of our game.

**Theorem 3.1**

*Assume that* $\hat{J}$ *has a minimum* $\bar{u}=({\bar{u}}^{\left(1\right)},{\bar{u}}^{\left(2\right)})$*. Then,* $\left({\bar{u}}^{\left(1\right)},{\bar{u}}^{\left(2\right)}\right)$ *is an NE of the game (3.1)–(3.2).*

**Proof.**

For all *u*^(1)^,*u*^(2)^∈*U*^(1)^×*U*^(2)^, we have $\hat{J}({\bar{u}}^{\left(1\right)},{\bar{u}}^{\left(2\right)})\leq \hat{J}(u^{\left(1\right)},u^{\left(2\right)})$, that is,

$$G_{1}({\bar{u}}^{\left(1\right)})+G_{2}({\bar{u}}^{\left(2\right)})+W({\bar{u}}^{\left(1\right)},{\bar{u}}^{\left(2\right)})\leq G_{1}(u^{\left(1\right)})+G_{2}(u^{\left(2\right)})+W(u^{\left(1\right)},u^{\left(2\right)}).$$

Now, set $u^{\left(1\right)}={\bar{u}}^{\left(1\right)}$ to obtain

$$G_{2}({\bar{u}}^{\left(2\right)})+W({\bar{u}}^{\left(1\right)},{\bar{u}}^{\left(2\right)})\leq G_{2}(u^{\left(2\right)})+W({\bar{u}}^{\left(1\right)},u^{\left(2\right)}),\;\forall u^{\left(2\right)}\in U^{\left(2\right)}.$$

This can be equivalently written as follows:

$$J_{2}({\bar{u}}^{\left(1\right)},{\bar{u}}^{\left(2\right)})\leq J_{2}({\bar{u}}^{\left(1\right)},u^{\left(2\right)}),\;\forall u^{\left(2\right)}\in U^{\left(1\right)}.$$

With the same reasoning, we also obtain

$$J_{1}({\bar{u}}^{\left(1\right)},{\bar{u}}^{\left(2\right)})\leq J_{1}(u^{\left(1\right)},{\bar{u}}^{\left(2\right)}),\;\forall u^{\left(1\right)}\in U^{\left(1\right)}.$$

Thus, the claim is proved. ▪

This theorem states that the existence of a minimum of $\hat{J}$ is a sufficient condition for a Nash equilibrium. On the other hand, this condition is not necessary, in the sense that there can be Nash equilibria that are not minima of $\hat{J}$.

Note that theorem 3.1 is the starting point to apply efficient tools from computational optimization to compute this equilibrium. This is the twofold purpose of the next section.

## 4. An optimal control problem

We recall that our NE $\bar{u}=({\bar{u}}^{\left(1\right)},{\bar{u}}^{\left(2\right)})$ satisfies the optimal control problem:

$$\hat{J}(\bar{u})\leq \hat{J}(u)\;\;\mathrm{for}\;\mathrm{all}\;u=(u^{\left(1\right)},u^{\left(2\right)})\in U^{\left(1\right)}\times U^{\left(2\right)},$$

which is the formulation of our optimization problem in reduced form. Thus, by explicitly stating the dependence of the PDFs on the control strategies, our optimization problem is explicitly given by

4.1 $$\begin{array}{r} \min\hat{J}(\;f^{\left(1\right)},f^{\left(2\right)},u^{\left(1\right)},u^{\left(2\right)}):=G_{1}(\;f^{\left(1\right)},u^{\left(1\right)})+G_{2}(\;f^{\left(2\right)},u^{\left(2\right)})+W(\;f^{\left(1\right)},f^{\left(2\right)}) \end{array}$$

4.2 $$\begin{array}{r} \partial_{t}f^{\left(1\right)}(x,t)-\frac{\sigma^{2}}{2}\sum_{i=1}^{D}\partial_{x_{i}x_{i}}^{2}f^{\left(1\right)}(x,t)+\sum_{i=1}^{D}\partial_{x_{i}}(b_{i}^{\left(1\right)}(x,t,u_{i}^{\left(1\right)}(t))f^{\left(1\right)}(x,t))=0, \end{array}$$

4.3 $$\begin{array}{r} f^{\left(1\right)}(x,0)=f_{0}^{\left(1\right)}(x), \end{array}$$

4.4 $$\begin{array}{r} \partial_{t}f^{\left(2\right)}(x,t)-\frac{\sigma^{2}}{2}\sum_{i=1}^{D}\partial_{x_{i}x_{i}}^{2}f^{\left(2\right)}(x,t)+\sum_{i=1}^{D}\partial_{x_{i}}(b_{i}^{\left(2\right)}(x,t,u_{i}^{\left(2\right)}(t))f^{\left(2\right)}(x,t))=0 \end{array}$$

4.5 $$\begin{array}{r} \text{and}\;\;f^{\left(2\right)}(x,0)=f_{0}^{\left(2\right)}(x), \end{array}$$

where flux zero boundary conditions are considered and we require that the strategies $u^{\left(p\right)}=(u_{1}^{\left(p\right)},\ldots ,u_{n}^{\left(p\right)})$, *p*=1,2, are in the following admissible set:

4.6 $$U^{\left(p\right)}=\{u\in H_{0}^{1}(0,T;R^{D})\;|\;u_{a}\leq u_{i}(t)\leq u_{b},\;i=1,\ldots ,D\text{ almost everywhere in }(0,T)\},$$

where $u_{a},u_{b}\in R,u_{a}<u_{b}$, and the reason for requiring a zero control at the beginning and the end of the time interval is discussed below.

Next, we discuss the existence of solutions to the optimal control problems (4.1)–(4.6). For this purpose, in the following lemma, we address the properties of the cost functional $\hat{J}$.

**Lemma 4.1**

*The objective functional* (*4.1*) *is sequentially weakly lower semicontinuous* (*w.l.s.c.*), *bounded from below, coercive on* *U*^(1)^×*U*^(2)^ *and it is Fréchet differentiable*.

The proof of this lemma is straightforward, once one recalls that the PDFs are non-negative functions.

The next result states the existence of an optimal control $\bar{u}$.

**Lemma 4.2**

*Assume that* $f_{0}=(\;f_{0}^{\left(1\right)},f_{0}^{\left(2\right)})\in H^{1}(\Omega )\times H^{1}(\Omega ),$ *that* $f_{0}^{\left(p\right)}$ *satisfies* (*2.6*) *and that the objective is given by* (*4.1*). *Then, there exist PDFs* ${\bar{f}}^{\left(p\right)}\in C([0,T];H^{1}(\Omega ))$, *p*=1,2, *and* $\bar{u}=({\bar{u}}^{\left(1\right)},{\bar{u}}^{\left(2\right)})\in U^{\left(1\right)}\times U^{\left(2\right)}$ *such that* $\bar{f}=(\;{\bar{f}}^{\left(1\right)},{\bar{f}}^{\left(2\right)})$ *solves the FP constraints* (*4.2*)–(*4.5*) *and* $\bar{u}$ *minimizes* $\hat{J}$ *in* *U*^(1)^×*U*^(2)^.

**Proof.**

Boundedness from below of $\hat{J}$ guarantees the existence of a minimizing sequence (*u*^*m*^)∈*U*^(1)^×*U*^(2)^. As *U*^(1)^×*U*^(2)^ is reflexive and $\hat{J}$ is sequentially w.l.s.c. and coercive in $H^{1}(0,T;R^{D})\times H^{1}(0,T;R^{D})$, this sequence is bounded. Therefore, it contains a weakly convergent subsequence (*u*^*m*_*l*_^) such that $u^{m_{l}}⇀\bar{u}$. Moreover, as the embedding *H*^1^(0,*T*)⊂⊂*C*(0,*T*) is compact, we have strong convergence $u^{m_{l}}\to \bar{u}$ in $C([0,T];R^{D})$.

In correspondence of the sequence (*u*^*m*_*l*_^), the solution of the FP equations results in the bounded sequence ( *f*^*m*_*l*_^)∈[*L*^2^(0,*T*;*H*^1^(*Ω*))∩*C*([0,*T*];*L*^2^(*Ω*))]^2^ while the sequence of the time derivatives, (∂_*t*_*f*^*m*_*l*_^), is bounded in [*L*^2^(0,*T*;*H*^−1^(*Ω*))]^2^. Therefore, both sequences converge weakly to $\bar{f}$ and $\partial_{t}\bar{f}$, respectively. Further, by the theorem of Aubin–Lions [25,26], we have that there exists a subsequence of ( *f*^*m*_*l*_^), which we denote by the same index, that converges strongly in [*L*^2^(0,*T*;*L*^2^(*Ω*))]^2^.

Concerning the terms ∇⋅(*b*(*x*,*t*,*u*)*f*), recall the structure of *b* given in (2.2) and the fact that *u* is only time dependent. Therefore, the sequence of the products *b*(⋅,⋅,*u*^*m*_*l*_^)*f*^*m*_*l*_^ converges strongly. Finally, considering these limiting sequences in the weak formulation of the FP problems, it follows that $\bar{f}$ corresponds to the solution of (4.2)–(4.5) with the control given by $\bar{u}$. Thus, the pair $\left(\bar{f},\bar{u}\right)$ minimizes the cost functional $\hat{J}$. ▪

Lemma 4.2 states the existence of a local optimal solution. However, the presence of possible symmetries in the formulation of the game, like invariance under exchange of players or geometrical symmetries, suggests that multiple Nash equilibria must exist.

A local minimum $\bar{u}$ of $\hat{J}$ is characterized by the first-order necessary optimality conditions given by $\langle \nabla \hat{J}(\bar{u}),v-\bar{u}\rangle \geq 0$ for all *v*∈*U*_*ad*_. We denote by 〈⋅,⋅〉 the $L^{2}(0,T;R^{D})\times L^{2}(0,T;R^{D})$ inner product (unless otherwise specified), and $\nabla \hat{J}(\bar{u})$ denotes the $L^{2}(0,T;R^{D})\times L^{2}(0,T;R^{D})$ gradient as the Riesz representative, in the $L^{2}(0,T;R^{D})\times L^{2}(0,T;R^{D})$ Hilbert space, of the derivative functional $d\hat{J}(\bar{u})$ evaluated at $\bar{u}$. We have $d\hat{J}(\bar{u})\cdot v=\langle \nabla \hat{J}(\bar{u}),v\rangle$.

It is well known that, in the framework of the adjoint method, the condition $\langle \nabla \hat{J}(\bar{u}),v-\bar{u}\rangle \geq 0$ results in the following optimality system, consisting of forward and backward FP problems and a variational inequality [27–29]. We have

4.7 $$\begin{array}{r} \partial_{t}f^{\left(1\right)}(x,t)-\frac{\sigma^{2}}{2}\sum_{i=1}^{D}\partial_{x_{i}x_{i}}^{2}f^{\left(1\right)}(x,t)+\sum_{i=1}^{D}\partial_{x_{i}}(b_{i}^{\left(1\right)}(x,t,u_{i}^{\left(1\right)}(t))f^{\left(1\right)}(x,t))=0, \end{array}$$

4.8 $$\begin{array}{r} f^{\left(1\right)}(x,0)=f_{0}^{\left(1\right)}(x), \end{array}$$

4.9 $$\begin{array}{r} \partial_{t}f^{\left(2\right)}(x,t)-\frac{\sigma^{2}}{2}\sum_{i=1}^{D}\partial_{x_{i}x_{i}}^{2}f^{\left(2\right)}(x,t)+\sum_{i=1}^{D}\partial_{x_{i}}(b_{i}^{\left(2\right)}(x,t,u_{i}^{\left(2\right)}(t))f^{\left(2\right)}(x,t))=0, \end{array}$$

4.10 $$\begin{array}{r} f^{\left(2\right)}(x,0)=f_{0}^{\left(2\right)}(x), \end{array}$$

4.11 $$\begin{array}{r} -\partial_{t}p^{\left(1\right)}(x,t)-\frac{\sigma^{2}}{2}\sum_{i=1}^{D}\partial_{x_{i}x_{i}}^{2}p^{\left(1\right)}(x,t)-\sum_{i=1}^{D}b_{i}^{\left(1\right)}(x,t,u_{i}^{\left(1\right)}(t))\partial_{x_{i}}p^{\left(1\right)}(x,t)+\rho f^{\left(2\right)}(x,t)=0, \end{array}$$

4.12 $$\begin{array}{r} p^{\left(1\right)}(x,T)=-\alpha V(x-x_{T}^{\left(1\right)}), \end{array}$$

4.13 $$\begin{array}{r} -\partial_{t}p^{\left(2\right)}(x,t)-\frac{\sigma^{2}}{2}\sum_{i=1}^{D}\partial_{x_{i}x_{i}}^{2}p^{\left(2\right)}(x,t)-\sum_{i=1}^{D}b_{i}^{\left(2\right)}(x,t,u_{i}^{\left(1\right)}(t))\partial_{x_{i}}p^{\left(2\right)}(x,t)+\rho f^{\left(1\right)}(x,t)=0 \end{array}$$

4.14 $$\begin{array}{r} \text{and}\;p^{\left(2\right)}(x,T)=-\alpha V(x-x_{T}^{\left(2\right)}). \end{array}$$

The variables *p*^(1)^ and *p*^(2)^ denote the Lagrange multipliers associated with the FP equations (4.7) and (4.9), respectively.

Further, the optimality condition is formally given by

4.15 $${\left(\nu u_{k}^{\left(p\right)}-\nu \frac{\partial^{2}u_{k}^{\left(p\right)}}{\partial t^{2}}-\int_{\Omega }\frac{\partial p^{\left(p\right)}}{\partial x_{k}}f^{\left(p\right)}\;dx,v_{k}^{\left(p\right)}-u_{k}^{\left(p\right)}\right)}_{L^{2}(0,T)}\geq 0\;\forall v_{k}^{\left(p\right)},\;k=1,\ldots ,D,$$

where $u_{k}^{\left(p\right)}$ represents the *k*th component of *u*^(*p*)^∈*U*_*p*_.

Note that in the optimality system, the following reduced *L*^2^-gradient components appear

4.16 $$\nabla_{u_{k}}\hat{J}(u)=\nu u_{k}-\nu \frac{\partial^{2}u_{k}}{\partial t^{2}}-\int_{\Omega }\frac{\partial p}{\partial x_{k}}f\;dx,\;k=1,\ldots ,D,$$

where the time Laplacian is meant in a distributional sense, and assuming that the last term in (4.16) is in *H*^−1^(0,*T*) and the control is zero at the initial and final times (controls switch on at *t*=0 and switch off at *t*=*T*), the solution of the gradient equation with homogeneous Dirichlet boundary conditions results in $u\in H_{0}^{1}(0,T;R^{D})$.

We wish to apply a gradient-based optimization scheme where the residual of (4.16) is used. For this purpose, we cannot use this residual directly for updating the control, because it is in *H*^−1^(0,*T*). Therefore, it is necessary to determine the corresponding reduced *H*^1^(0,*T*) gradient. This is done based on the following fact:

$${\left\langle \nabla \hat{J}{\left(u\right)}_{H^{1}},\varphi \right\rangle }_{H^{1}((0,T);R^{D})}={\left\langle \nabla \hat{J}(u),\varphi \right\rangle }_{L^{2}((0,T);R^{D})}\;\text{for all }\varphi \in H^{1}((0,T);R^{D}).$$

Using the definition of the *H*^1^ inner product and integrating by parts, we have that the *H*^1^ gradient can be obtained by solving the following boundary-value problem:

4.17 $$-\Delta (\nabla_{u_{k}}\hat{J}{\left(u\right)}_{H^{1}})+(\nabla_{u_{k}}\hat{J}{\left(u\right)}_{H^{1}})=\nabla_{u_{k}}\hat{J}(u)\;\text{in }(0,T)$$

and

4.18 $$(\nabla_{u_{k}}\hat{J}{\left(u\right)}_{H^{1}})=0\;\text{ on }\partial (0,T),$$

where *k*=1,…,*D*, and *Δ* denotes the Laplace operator in time.

The solution to this problem provides the appropriate gradient to be used in a gradient update of the control that includes projection to satisfy the given control constraints.

## 5. Numerical experiments

We are now ready to consider four different test cases to discuss the validity of our FP-Nash approach to pedestrian avoidance and test the efficiency and robustness of our optimization set-up for determining the NE pair $\left({\bar{u}}^{\left(1\right)},{\bar{u}}^{\left(2\right)}\right)$ for the differential game (3.3)–(3.4). To assess the ability of our approach to model real avoidance dynamics, we compare our results to field cognitive psychology studies involving experiments with humans [16–18].

The motion of the pedestrians/players are represented by the motion of their PDFs. In the plots, the trajectories of the players depict the trajectories of the mean of the position computed using their respective PDFs. The initial density for the players are defined as follows:

5.1 $$f_{0}(x)=\hat{C}\;e^{-\{{\left(x_{1}-A_{1}\right)}^{2}-{\left(x_{2}-A_{2}\right)}^{2}\}/0.5},$$

where (*A*_1_,*A*_2_)=*x*_*t*_(0) is the starting point of the pedestrians *X*, and $\hat{C}$ is a normalization constant such that

$$\int_{\Omega }f_{0}(x)\;dx=1$$

and *x*=(*x*_1_,*x*_2_). The terminal potentials for both the pedestrians are defined as

$$V(x-x_{T}^{\left(p\right)})={\left(x-x_{T}^{\left(p\right)}\right)}^{2},\;p=1,2,$$

where $x_{T}^{\left(p\right)}$ is the terminal position of the *p*th pedestrian. The parameter *α* is set to be 100 and *ν*=1.

The differential game consists in determining NE strategies $\left(u_{{\;}_{\mathrm{NE}}}^{\left(1\right)},u_{{\;}_{\mathrm{NE}}}^{\left(2\right)}\right)$ for the players A and B, respectively, such that they avoid interaction with each other while maintaining their respective drifts in order to reach the terminal point. The intensity of avoidance is dependent on the factor *ρ*.

We solve the optimal control problem (4.1)–(4.6), which gives a NE, using a gradient-based optimization scheme as in [21].

In the following, we use the notations *D*^(*p*)^ and *A*^(*p*)^ to define, respectively, (D)eparture and (A)rrival locations of pedestrian A for *p*=1, and B for *p*=2.

### 5.1. Test case I: Huber-135

**Figure 2.**
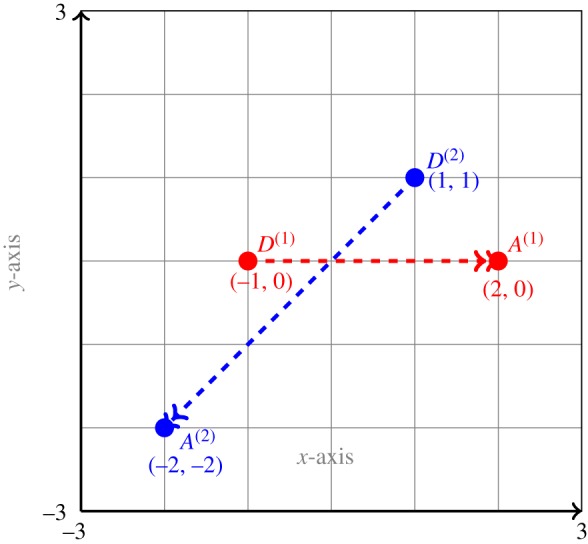
Settings for the game described in test case Huber-135.

The test case Huber-135 corresponds to the experiment in [16] where trajectories of agents A and B form an angle of 135°. The computational setting is the following: we consider the motion of A and B in a square domain *Ω*=[−3,3]×[−3,3] for a time interval [0,*T*] for *T*=3. Player A starts its motion from the departure point *D*^(1)^=(−1,0) and player B starts its motion from the point *D*^(2)^=(1,1). The drift *v*^(1)^ for player A is (1,0), thus it moves along the *x*-axis. The drift *v*^(2)^ for player B is (−1,−1) and, thus, it moves down diagonally. The arrival position of player A is *A*^(1)^=(2,0) and for player B is *A*^(2)^=(−2,−2). The spatial and the temporal domains are divided into 50 uniformly distributed subintervals. The settings for the game are shown in figure 2.

**Figure 3.**
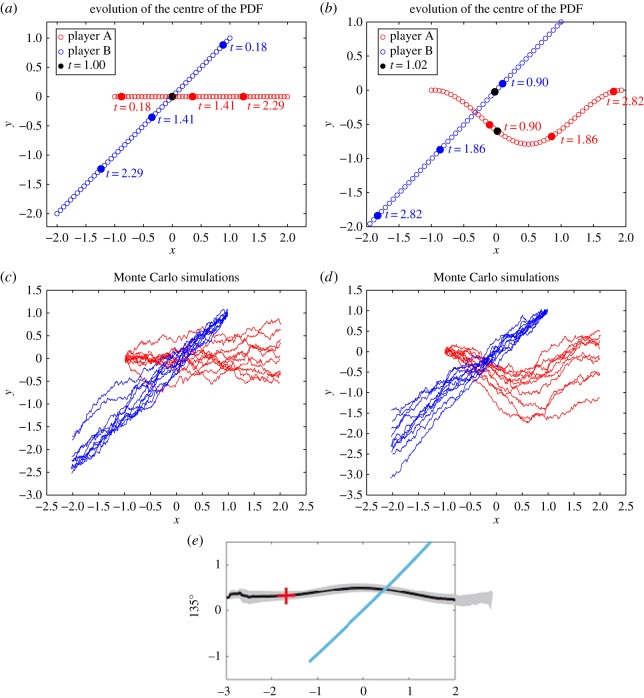
Test case I: (*a*,*b*) the plots of the mean of the PDFs for *ρ*= 0.01,200, respectively; (*c*,*d*) the results of Monte Carlo simulation for *ρ*=0.01,200, respectively and (*e*) the results of the corresponding real experiment from [16].

 Figure 3*a*,*b* shows the plots of the pedestrian mean positions for *ρ*=0.01, 200, respectively. We can see that, for *ρ*=0.01, the two players meet at time *t*=1.0 as denoted by the black dot. In the case *ρ*=200, pedestrian A goes around pedestrian B to avoid intersection as is shown from the position of the players at time *t*=1.0 with the black dot. Figure 3*c*,*d* show the results of the corresponding Monte Carlo SDEs simulation for *ρ*=0.01, 200, respectively. In both the cases, we see that the two players reach their final target. This is comparable with the results of the corresponding experiment with humans presented in [16], which is shown in figure 3*e*. However, note that there was no game involved in [16] as one of the experiment participants was a non-reactive interferer. We remark that, in the present test case, player B acts precisely as a non-reactive agent in the NE.

### 5.2. Test case II: Turnwald 1C-A3

**Figure 4.**
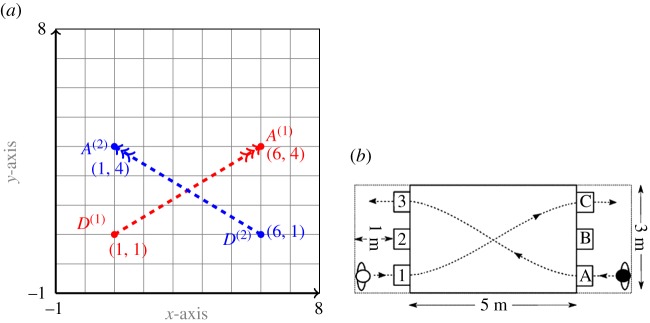
Settings for the Turnwald game described in test case Turnwald 1C-A3: (*a*) the computational setting and (*b*) the real experiment setting excerpt from [18].

In the next test case, we consider an experiment from [18]. Two participants A and B are asked to walk from given initial to final points, the latter information being known to both players. In the present case, 1C-A3 means player A goes from ‘1 to *C*’, and player B goes from ‘*A* to 3’ as shown in figure 4*b*. In our computational setting, the motion of A and B is in a square domain *Ω*=[−1,8]×[−1,8] for a time interval [0,*T*] for *T*=5. Pedestrian A starts its motion from the departure point *D*^(1)^=(1,1) and player B starts its motion from the point *D*^(2)^=(6,1). The arrival position of player A is *A*^(1)^=(6,4) and that for player B is *A*^(2)^=(1,4). The spatial and the temporal domains are divided into 50 uniformly distributed subintervals. The settings for the game are shown in figure 4.

**Figure 5.**
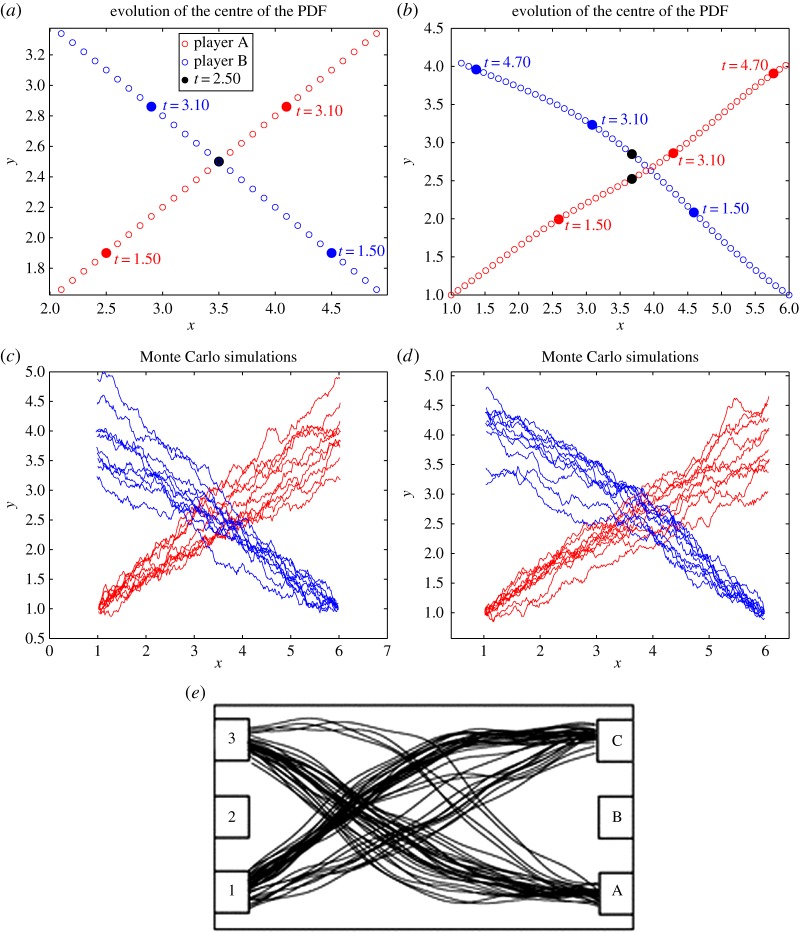
Test case II: (*a*,*b*) the zoomed-in plots of the mean positions with *ρ*=0.01,200, respectively. Left: motion for *ρ*=0.01; (*c*,*d*) the results of Monte Carlo simulation for *ρ*=0.01,200, respectively and (*e*) the results of the experiment in [18].

 Figure 5*a*,*b* shows the zoomed-in plots of the mean of the PDFs for *ρ*=0.01,200, respectively. We note that, for *ρ*=0.01, the two pedestrians meet at time *t*=2.5 as denoted by the black dot. In the case *ρ*=200, players A and B move around each other to avoid intersection as is shown by their positions at time *t*=2.5 with a black dot. Figure 5*c*,*d* shows the Monte Carlo simulations for *ρ*=0.01,200, respectively. For *ρ*=200, we see that, around the region where trajectories cross, the layout of the Monte Carlo simulation is comparable with the one from [18] shown in figure 5*e*. Note that, in figure 5*e*, trajectories show features (slopes) different from those of figure 5*d*. In our opinion, this is due to the fact that, owing to availability of visual information, the players can and indeed play a dynamic game, not a static one, and adapt their strategies by integrating the observed visual information.

**Figure 6.**
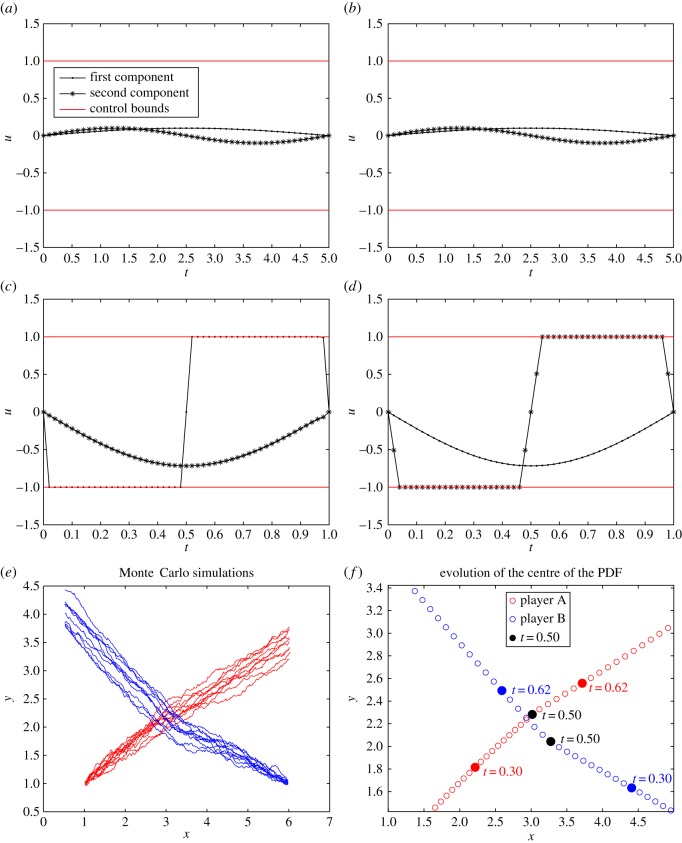
Test case II: (*a*,*b*) the plots of the first and second components of the controls *u*_1_ and *u*_2_, respectively, for the case when test case II is performed in the time interval [0,5]. (*c*,*d*) The plots of the components of the controls *u*_1_ and *u*_2_, respectively, for the case when test case II is performed in the time interval [0,1]. (*e*) The results of Monte Carlo simulation. (*f*) The zoomed-in plots of the mean positions with *ρ*=200 in the case when *T*=1.

The results of test case II correspond to the choice of *T*=5. In this case with a relatively long time horizon, the resulting controls, which are depicted in the figure 6*a*,*b*, are able to perform avoidance while the corresponding control bounds (represented by horizontal lines) remain inactive. However, choosing a shorter time horizon, e.g. *T*=1, a much greater effort for avoidance is required and, in this case, the larger controls reach their bounds as shown in the figure 6*c*,*d*. Nevertheless, also in the case of a shorter time horizon, avoidance takes place as shown in figure 6*f*.

### 5.3. Test case III: Turnwald 1C-B2

**Figure 7.**
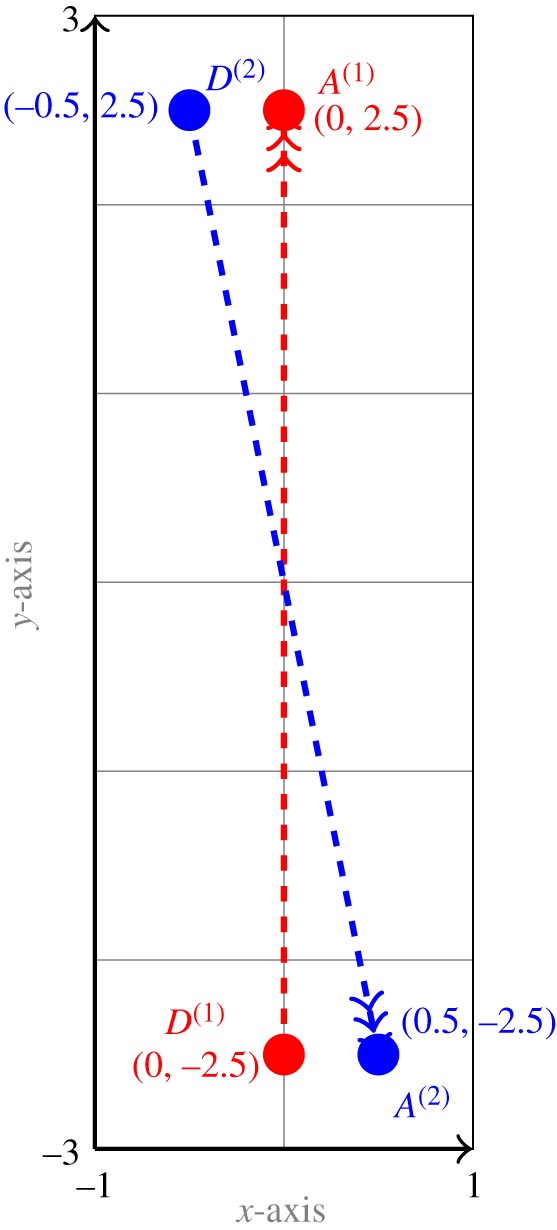
Settings for the Turnwald game described in test case Turnwald 1C-B2.

**Figure 8.**
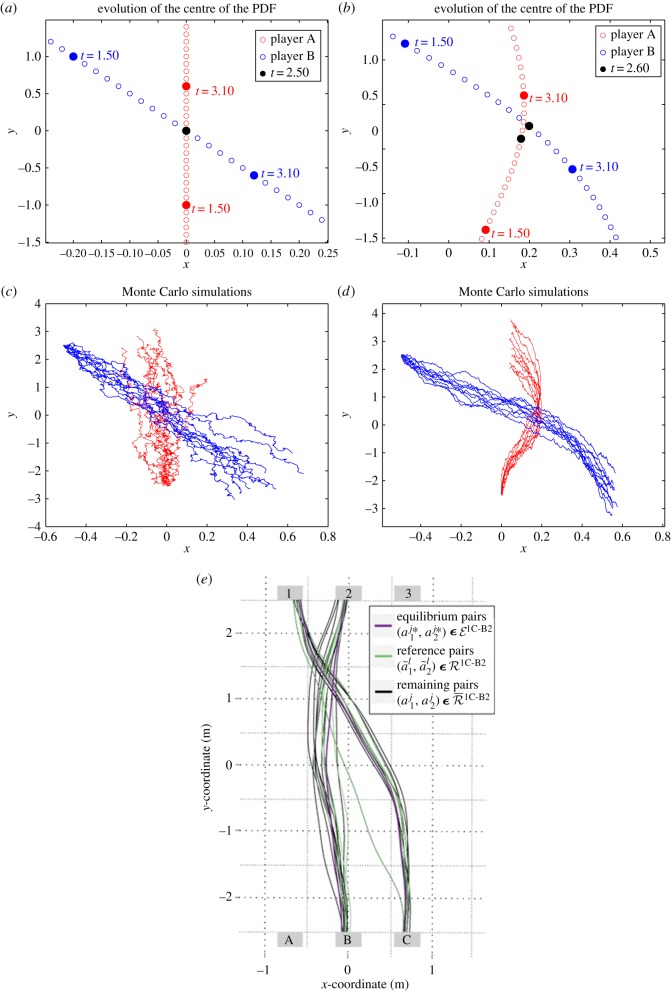
Test case III: (*a*,*b*) the zoomed-in plots of the mean of the PDFs for *ρ*=0.01,150, respectively. Left: motion for *ρ*=0.01. (*c*,*d*) The results of Monte Carlo simulations for *ρ*=0.01,150, respectively. (*e*) The results of Monte Carlo simulation for the experimental results in [18].

In the next test case, we consider another experiment from [18]. In this case, player A goes from ‘*B* to 2’ and player B goes from ‘1 to *C*’ as shown in figure 7. In the computational setting shown in figure 8*e*, the motion of A and B is in the domain *Ω*=[−1,1]×[−3,3] for a time interval [0,*T*] for *T*=5. Player A starts moving from the departure point *D*^(1)^=(0,−2.5) and player B starts its motion from the point *D*^(2)^=(−0.5,2.5). The terminal position of player A is *A*^(1)^=(0,2.5) and that for player B is *A*^(2)^=(0.5,−2.5).

 Figure 8*a*,*b* shows the zoomed-in plots of the mean pedestrian positions for *ρ*=0.01,150, respectively. We note that, for *ρ*=0.01, the two players meet at time *t*=2.5 as denoted by the black dot. In the case *ρ*=150, players A and B move around each other to avoid intersection as is shown by their positions at time *t*=2.6 with a black dot. Figure 8*c*,*d* shows the results of Monte Carlo simulation for *ρ*=0.01,150, respectively.

With respect to the previous experiment, Turnwald 1C-A3, we see that, for *ρ*=150, the results of Monte Carlo simulation show even more geometric similarities with the ones resulting from the human experiments in [18] as shown in figure 8*e*. However, as in the previous test case, we observe that our NE trajectories still exhibit differences with respect to the experiment with humans. This is due, in our opinion, to the same reasons discussed for Turnwald 1C-A3. Indeed, the participants play a dynamic game, and adapt their strategies according to the available visual information they get dynamically from the others.

For instance, for the two Turnwald real experiments, one of the players observes that the other player initiates a straight motion and then reacts by deviating from the straight line, following a path which ensures sufficiently large minimal predicted distance (MPD), as suggested in [17], but then increasing the cost of the strategy. In our Nash game, the control over the cost of the strategies forces the trajectories of the players to deviate less, resulting in a smaller MPD.

### 5.4. Test case IV: Gait and Posture

**Figure 9.**
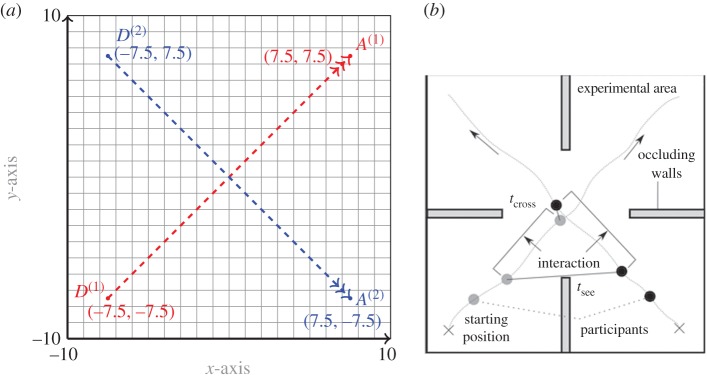
Computational (*a*) and experimental (*b*) settings for the Gait and Posture game.

In the final test case, we consider an experiment from [17], which we call the ‘Gait and Posture’ experiment. In this experiment, two participants, separated by blankets, are asked to move across the experimental area, to reach a prescribed location (figure 9*b*). In our computational setting shown in figure 9*a*, the motion of two players A and B is in a square domain *Ω*=[−10,10]×[−10,10] for a time interval [0,*T*] for *T*=1. Player A starts its motion from the departure point *D*^(1)^=(−7.5,−7.5) and player B starts its motion from the point *D*^(2)^=(−7.5,7.5). The terminal position of player A is *A*^(1)^=(7.5,7.5) and that of player B is *A*^(2)^=(7.5,−7.5).

**Figure 10.**
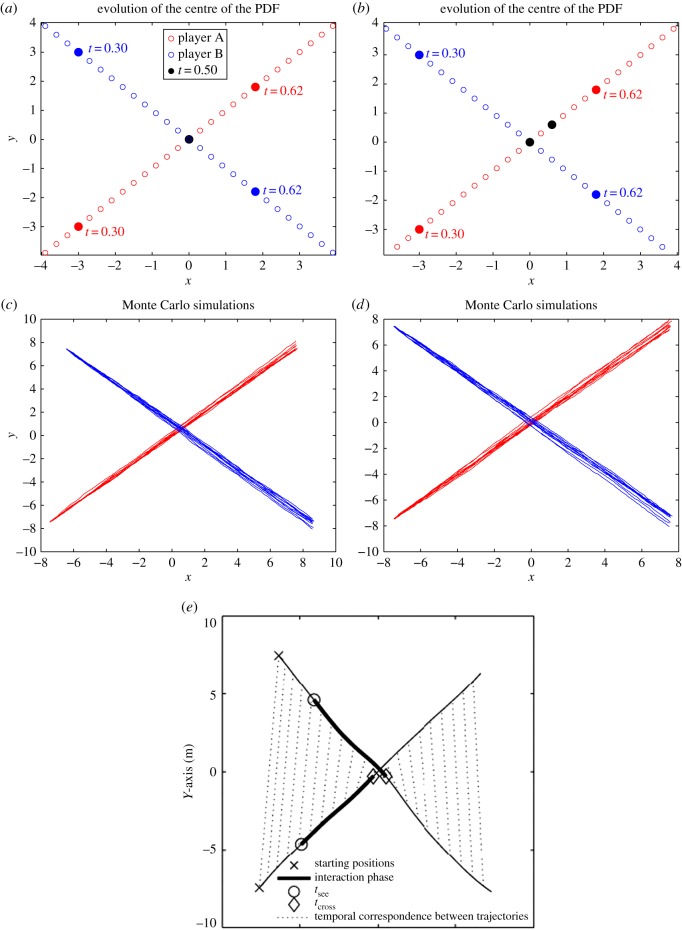
Test case IV: (*a*,*b*) the zoomed-in plots of the mean of the PDFs for *ρ*=0.01,200, respectively. (*c*,*d*) The results of Monte Carlo simulation for *ρ*=0.01,200, respectively. (*e*) The trajectories for the experimental results in [17].

 Figure 10*a*,*b* shows the zoomed-in plots of the mean of the PDFs for *ρ*=0.01,200, respectively. We note that, for *ρ*=0.01, the two pedestrian meet at time *t*=0.5 as denoted by the black dot. In the case *ρ*=200, player A moves faster near the time of intersection *t*=0.5 to avoid intersection with player B. After avoiding intersection, player A goes slower to reach its terminal target.

We remark that figure 10*b*,*e* is in very good accordance with respect to the trajectories followed by the real pedestrian as well as with respect to the velocity profiles. By observing the ‘temporal correspondence between trajectories’ as in figure 10*e*, we note that one pedestrian moves faster near the time of intersection ‘*t*_cross_’ to avoid intersection with the other pedestrian. After avoiding intersection, the first player goes slower to reach its terminal target. In comparison to the previous Turnwald experiments, which involved early visual information weakening the static game assumption, in this case the real experiment is set up as a static (or blind or simultaneous) Nash game, and the results of our computational model are strikingly similar to the results of the real experiment.

## 6. Conclusion

A new approach to modelling pedestrian's avoidance dynamics based on a FP-Nash game framework was presented and investigated theoretically and numerically. This approach attempts to explain why the observed dynamics arise, while classical phenomenological approaches prescribe how the dynamics should be.

Based on FP equations, a Nash differential game was formulated where the game strategies represent controls aiming at avoidance by minimizing appropriate collision cost functionals. The existence of a Nash equilibria solution was proved and characterized as a solution to an optimal control problem. The resulting FP-Nash computational strategy for pedestrian motion was successfully benchmarked with results of real experiments from cognitive psychology studies. The proposed FP-Nash approach represents a powerful new paradigm for the theoretical investigation and computational simulation of differential games with non-convex cost functionals, and it provides a very rich framework to model a large class of processes involved in pedestrian motion.

## Supplementary Material

Matlab package for the Turnwald-1C-A3 experiment

## Supplementary Material

Matlab package for the Gait and Posture experiment

## Data accessibility

Data concerning results of real human experiments are available in [16–18]. The remaining data can be found within the manuscript. The electronic supplementary material provides the Matlab codes used for the simulations and the necessary input.
